# Factors influencing the quality and functioning of oncological multidisciplinary team meetings: results of a systematic review

**DOI:** 10.1186/s12913-022-08112-0

**Published:** 2022-06-27

**Authors:** Janneke E. W. Walraven, Olga L. van der Hel, J. J. M. van der Hoeven, Valery E. P. P. Lemmens, Rob H. A. Verhoeven, Ingrid M. E. Desar

**Affiliations:** 1 Department of Medical Oncology, Radboud University Medical Center, Postbus 9101, huispost 415, Nijmegen, 6500 HB The Netherlands; 2 Department of Research, Netherlands Comprehensive Cancer Organization, Goldebaldkwartier 419, Utrecht, 3511 DT The Netherlands; 3 Department of Medical Oncology, Amsterdam UMC, University of Amsterdam, Cancer Center AmsterdamMeibergdreef 9, Amsterdam, 1105 AZ The Netherlands

**Keywords:** Multidisciplinary team meeting, Quality, Team culture, Decision making, Education, Evaluation

## Abstract

**Background:**

Discussing patients with cancer in a multidisciplinary team meeting (MDTM) is customary in cancer care worldwide and requires a significant investment in terms of funding and time. Efficient collaboration and communication between healthcare providers in all the specialisms involved is therefore crucial. However, evidence-based criteria that can guarantee high-quality functioning on the part of MDTMs are lacking. In this systematic review, we examine the factors influencing the MDTMs’ efficiency, functioning and quality, and offer recommendations for improvement.

**Methods:**

Relevant studies were identified by searching Medline, EMBASE, and PsycINFO databases (01–01-1990 to 09–11-2021), using different descriptions of ‘MDTM’ and ‘neoplasm’ as search terms. Inclusion criteria were: quality of MDTM, functioning of MDTM, framework and execution of MDTM, decision-making process, education, patient advocacy, patient involvement and evaluation tools. Full text assessment was performed by two individual authors and checked by a third author.

**Results:**

Seventy-four articles met the inclusion criteria and five themes were identified: 1) MDTM characteristics and logistics, 2) team culture, 3) decision making, 4) education, and 5) evaluation and data collection. The quality of MDTMs improves when the meeting is scheduled, structured, prepared and attended by all core members, guided by a qualified chairperson and supported by an administrator. An appropriate amount of time per case needs to be established and streamlining of cases (i.e. discussing a predefined selection of cases rather than discussing every case) might be a way to achieve this. Patient centeredness contributes to correct diagnosis and decision making. While physicians are cautious about patients participating in their own MDTM, the majority of patients report feeling better informed without experiencing increased anxiety. Attendance at MDTMs results in closer working relationships between physicians and provides some medico-legal protection. To ensure well-functioning MDTMs in the future, junior physicians should play a prominent role in the decision-making process. Several evaluation tools have been developed to assess the functioning of MDTMs.

**Conclusions:**

MDTMs would benefit from a more structured meeting, attendance of core members and especially the attending physician, streamlining of cases and structured evaluation. Patient centeredness, personal competences of MDTM participants and education are not given sufficient attention.

**Supplementary information:**

The online version contains supplementary material available at 10.1186/s12913-022-08112-0.

## Introduction

In a context of increasingly complex multidisciplinary cancer treatments and centralisation of cancer care, the role of multidisciplinary team meetings (MDTMs) is growing in importance. In these, usually weekly, meetings, all healthcare providers involved discuss patient cases to formulate the diagnostic or therapeutic strategy [1–4]. In 1995, the Calman-Hine report set out principles regarding the organisation and structure of high-quality multidisciplinary care [5]. These were further developed by the British National Cancer Action Team in 2010 [5, 6]. MDTMs were set up in accordance with these principles worldwide and today constitute the standard of care [7–10]. Several national guidelines, as in the Netherlands [11], UK [8, 12], France [13], USA [14] and Australia [15], require discussion of nearly all cancer patients in an MDTM prior to initial treatment, despite a lack of strong evidence supporting survival benefit or improved quality of life for patients [16–18]. Worldwide, there are no evidence-based criteria guaranteeing high-quality MDTM functioning. In this review we explore the factors that influence the efficiency, functioning and quality of MDTMs. The impact of MDTMs on clinical outcomes in terms of survival or quality of life is beyond the scope of this review.

## Methods

We conducted a systematic review to identify factors that influence the efficiency, performance and quality of MDTMs. Articles written in English and published between 1–1-1990 and 9–11-2021 in the following electronic databases: Medline, Embase, PsychInfo were included. The search terms that were used were different descriptions of ‘oncological multidisciplinary team meeting’ in title or abstract and ‘neoplasm’ as MeSH or Emtree term. The search string is presented in Supplement A. In this generic search string, all articles on oncological MDTMs were collected and checked for eligibility based on the in- and exclusion criteria. The inclusion criteria were: full-text original research articles and any of the following subjects: quality of MDTM, functioning of MDTM, framework and execution of MDTM, decision-making process, education, patient advocacy, patient involvement, and evaluation tools. The exclusion criteria were: no original research article, non-oncological or paediatric MDTMs and articles that fully addressed any of the following topics: impact on patient outcomes or outcomes of the MDTMs regarding medical endpoints (e.g. change of treatment strategy, revised diagnosis), costs of MDTMs, results of MDTMs (e.g. proportion of patients discussed) and implementation of MDTMs.

Title and abstract were independently screened for relevance by two authors (JW and OvdH) based on the in- and exclusion criteria. In cases of discrepancy in judgement, a third author (ID) was consulted. Articles that appeared to meet the research question were assessed by full-text review, again by two authors (JW and OvdH) independently, and checked by a third author (ID). Full-text papers were checked for eligibility and quality. After agreement was reached on the articles to be included, JW extracted the relevant data from these studies and stored this data on the computers of the hospital where the authors work. The extracted data was reviewed by ID. Due to the many different study designs of the included articles, full data coding was not feasible. Therefore the data was classified by an inductive process (JW and ID) into themes and categories, which were examined by OvdH and KvdH. It should be noted that given the lack of formal evidence-based criteria that guarantee high-quality functioning of an MDTM, assumptions were sometimes made (e.g. we assumed that an incomplete team during the MDTM presents a risk for quality).

## Results

**Fig. 1:**
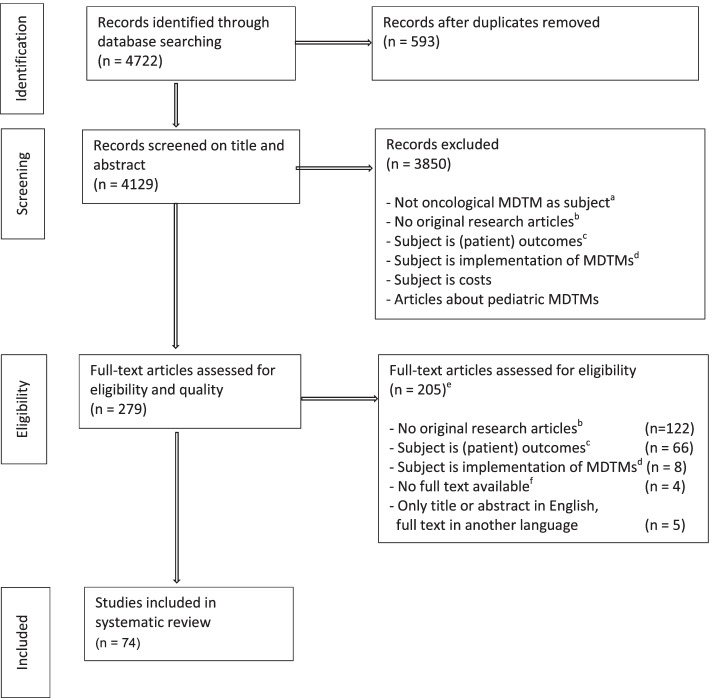
Study selection process. ^a^ Case reports, conference abstracts, cancer care, interdisciplinary or multidisciplinary treatment, multidisciplinary team, multidisciplinary management, multidisciplinary recommendation, multidisciplinary clinics, molecular tumour board. ^b^ Letters, correspondences, author reply, comments, descriptions, reports, orals, editorials, experiences, perspectives, opinions, study protocols, implementation protocols, overviews, reviews and, systematic reviews. ^c^ Subjects were outcomes on survival, diagnostics, pathology reports, radiological information, trial recruitment, comorbidity, adherence to guidelines, and adherence to MDTM recommendation, time to treatment. ^d^ Articles about whether or not MDTMs should be implemented in daily practice. ^e^ When there was a discrepancy between 2 researchers (OvdH, JW) as to whether or not the article should be included, a third researcher (ID) was consulted. After discussion between the three researchers, the final decision was made. ^f^ Four articles published between 1995 and 2005

Of an initial number of 4129 articles, 74 met the inclusion criteria (Fig. 1). Five themes with factors influencing functioning were identified: 1) MDTM characteristics and logistics, 2) team culture, 3) decision making, 4) education and 5) evaluation and data collection. Within these five themes a total of 10 subcategories were identified. In Table 1, all included studies are summarised, including the themes and subcategories they cover. In Table 2, recommendations are provided based on the results.

**Table 1:** Summary of included articles and representation of themes involved^c^

Reference	Study design	Study characteristics	Themes and categories				
			**MDTM characteristics and logistics**^**a**^	**Team culture**	**Decision making**^**b**^	Education	Evaluation and data collection
Bate J. et al., 2019 [19]	Qualitative study	Type of MDTM: Ewing sarcoma (ES) Data collection method: survey and focus group *N* = 15 survey responders and 10 focus group participants of ES patients Period: October 2016 – January 2017 Country: United Kingdom			* I		
Bohmeier B. et al., 2021 [20]	Qualitative study	Type of MDTM: breast cancer, gynaecological cancer Data collection method: semi-structured interviews *N* = 30 interviews to MDT-members (RR 28%) Period: June 2017 – May 2020 Country: Germany	* E		* HI		
Bolle S. et al., 2019 [21]	Prospective observational study	Type of MDTM: colorectal cancer Data collection method: MDTM observation *N* = 741 patient cases in 30 MDTMs observed Period: - Country: The Netherlands			* HI		
Butow P. et al., 2007 [22]	Qualitative study	Type of MDTM: breast cancer Data collection method: survey *N* = 640 surveys to surgeons, nurses, patient advocates (RR 78%) Period: - Country: Australia	* E				
Chaillou D. et al., 2019 [23]	Qualitative study	Type of MDTM: head and neck cancer Data collection method: survey *N* = 3 surveys in 54 patients (RR 63%) Period: December 2016 – March 2017 Country: France	* E				
Choy E. et al., 2007 [24]	Qualitative study	Type of MDTM: breast cancer Data collection method: survey and interviews *N* = 30 patients participated at MDTM *N* = 17 participating health professionals Period: 2006 Australia			* I		
Davison A. et al., 2004 [25]	Retrospective observational study	Type of MDTM: lung cancer Data collection method: analyse database *N* = 28 telemedicine MDTMs *N* = 62 patients Period: November 2000 – November 2001 Country: United Kingdom	* B				
Delaney G. et al., 2004 [26]	Observational study	Type of MDTM: breast cancer Data collection method: *N* = 27 breast cancer specialists; survey before (RR 59%) and after (RR 62%) start videoconferencing Period: February 2000 – June 2000 Country: Australia	* B	*			
Devitt B. et al., 2010 [27]	Qualitative study	Type of MDTM: not specified Data collection method: focus groups *N* = 4 focus groups, in total 23 MDT-members Period: - Country: Australia	* E	*	* H	*	
Dew K. et al., 2015 [12]	Prospective observational study + qualitative study	Type of MDTM: breast cancer, lung cancer, upper-gastro-intestinal cancers, colorectal cancer Data collection method: MDTM recording and content analysis *N* = 10 MDTMs audio recorded, 106 patients discussed Period: - Country: New Zealand			* H		
Díez J. et al., 2019 [28]	Qualitative study	Type of MDTM: all Data collection method: survey *N* = 71 MDTM leaders Period: February 2017 – February 2018 Country: Spain				*	
Evans L. et al., 2019 [29]	Qualitative study	Type of MDTM: all Data collection method: Survey and self-assessment *N* = 180 surveys / 19 MDTs (1^st^cycle), 118 surveys / 12 MDTs (2^nd^ cycle) completed a self-assessment matrix Period: 2017 and 2018 Country: Australia					*
Evans L. et al., 2021 [30]	Qualitative study	Type of MDTM: all Data collection method: Survey and self-assessment *N* = 121 surveys (1^st^cycle, RR 52%), 118 surveys (2^nd^ cycle, RR 52%), 146 surveys (3^rd^ cycle, RR 65%) completed a self-assessment matrix Period: 2017, 2018, 2019 Country: Australia					*
Fahim C. et al., 2020 [31]	Qualitative study	Type of MDTM: all Data collection method: Interviews and focus groups *N* = 21 interviews with MDT-members *N* = 18 focus group participants (MDT-members) Period: April 2016 – July 2017 Country: Canada	* ABCDG	*	* H	*	
Fahim C. et al., 2020 [32]	Prospective observational study	Type of MDTM: all Implementation of ‘Knowledge Training strategy’ Data collection method: MDTM observation *N* = 143 cases / 23 MDTMs (before phase) and 260 cases / 35 MDTMs (after phase) Period: - Country: Canada			* H		*
Farrugia D. et al., 2015 [33]	Retrospective observational study	Type of MDTM: breast cancer Data collection method: analyse database RQRS = Rapid quality reporting system = web-based standardized documentation template based on national guidelines *N* = 140 patients in cohort of 10 weeks before start RQRS *N* = 142 patients in cohort 10 weeks after start RQRS Period: July – October 2013 Country: United States of America	* G				
Field K. et al., 2010 [34]	Qualitative study	Type of MDTM: neuro-oncology Data collection method: survey *N* = 16 MDT-members (RR 100%) Period: July 2009 Country: Australia	* FG				
Gandamihardja T. et al., 2019 [35]	Prospective observational study	Type of MDTM: breast cancer Data collection method: MDTM observation *N* = 346 cases Period:- Country: United Kingdom	* F	*			*
Gatcliffe T. et al., 2008 [36]	Prospective observational study	Type of MDTM: gynaecological Data collection method: MDTM recording *N* = 52 MDTMs Period: May 2002 – August 2003 Country: United States of America				*	
Hahlweg P. et al., 2015 [37]	Prospective observational study and qualitative study	Type of MDTM: all Data collection method: MDTM observation and content analysis and descriptive statistics *N* = 15 MDTMs observed Period: November – December 2013 Country: Germany	* G		* HI	*	
Hahlweg P. et al., 2017 [38]	Cross-sectional observational study	Type of MDTM: all Data collection method: MDTM observation *N* = 249 cases, 29 MDTMs Period: - Country: Germany			* I		
Hamilton D. et al., 2016 [39]	Prospective observational study + qualitative study	Type of MDTM: head and neck cancers Data collection method: MDTM observation and ethnographic analyses *N* = 35 MDTMs and 37 MDT clinics observed; audio recorded and transcribed verbatim *N* = semi structured interviews with 20 patients and 9 MDTM staff members Period: - Country: United Kingdom			* I		
Harris J. et al., 2014 [40]	Prospective observational study + qualitative study	Type of MDTM: all Data collection method: MDTM observation and interviews *N* = 20 MDTMs observed N = 64 team members, 19 peer observers interviews Period: - Country: United Kingdom					*
Harris J. et al., 2016 [41]	Prospective observational study	Type of MDTM: all Data collection method: MDTM observation *N* = 10 MDTMs, 13 health service staff observers, 1 clinical and 1 non-clinical observer Period: - Country: United Kingdom					*
Hoinvile L. et al., 2019 [42]	Qualitative study	Type of MDTM: all Data collection method: survey *N* = 1220 MDT-members Period: March – May 2017 Country: United Kingdom	* F				
Huizen van S., et al. 2019 [43]	Prospective observational study + qualitative study	Type of MDTM: head and neck cancer Data collection method: MDTM observation and semi-structured interviews *N* = 6 interviews to MDT-members, *N* = 336 cases observed Period: - Country: The Netherlands	* F				
Jalil R. et al., 2012 [44]	Qualitative study	Type of MDTM: all Data collection method: survey *N* = 265 MDT-coordinators Period: - Country: United Kingdom	* G				
Jalil R. et al., 2013 [45]	Qualitative study	Type of MDTM: urology and gastroenterology Data collection method: interviews *N* = 22 MDTM members Period: - Country: United Kingdom	* BCE	*	* HI		
Jalil R. et al., 2014 [46]	Prospective observational study	Type of MDTM: urology Data collection method: MDTM observation *N* = 683 case discussions, 127 video recorded case discussions Period: - Country: United Kingdom					*
Janssen A. et al., 2018 [47]	Prospective observational study + qualitative study	Type of MDTM: all Data collection method: MDTM observation and semi-structured interviews *N* = 7 teams, 43 MDTMS *N* = 15 interviews Period: - Country: Australia	* BCG		* H	*	*
Jenkins V. et al., 2001 [48]	Qualitative study	Type of MDTM: breast cancer Data collection method: survey *N* = 64 MDT-members (RR 91%) Period: - Country: United Kingdom		*	* H		
Johnson C. et al., 2017 [49]	Qualitative study	Type of MDTM: All Data collection method: survey *N* = 3 MDTMs Period: - Country: Australia					*
Kunkler I. et al., 2006 [50]	Qualitative study	Type of MDTM: breast cancer Data collection method: survey N = 68 participants to Group behavior Inventory (GBI) survey: face-to-face GBI (RR 65%), post telemedicine GBI (RR 51%) Period: - Country: United Kingdom		*	*H		
Lamb B. et al., 2011 [51]	Qualitative study	Type of MDTM: all Data collection method: Semi-structured interviews *N* = 19 interviews with clinicians Period: October 2009 – April 2010 Country: United Kingdom	*ABCD	*			
Lamb B. et al., 2011 [52]	Prospective observational study + qualitative study	Type of MDTM: all Data collection method: MDTM observation and survey *N* = 164 cases observed, 67 surveys to MDT-members (RR 70%) Period: September – November 2009 Country: United Kingdom			* H		*
Lamb B. et al., 2012 [53]	Qualitative study	Type of MDTM: All Data collection method: Survey *N* = 175 MDTM members Period: October 2010 – April 2011 Country: United Kingdom	* B		* H		
Lamb B. et al., 2013 [54]	Longitudinal observational study	Type of MDTM: urology Data collection method: *N* = 36 MDTMs, 1421 patients Multicomponent intervention: MDTM checklists before start, team training and written guidance Period: December 2009 – April 2011 Country: United Kingdom	* BC		* H		
Lamb B. et al., 2013 [55]	Prospective observational study	Type of MDTM: urology Data collection method: MDTM observation *N* = 7 MDTMs, assessing 298 cases Period: December 2009 – January 2010 Country: United Kingdom	* CDF		* H		
Lamb B. et al., 2013 [56]	Qualitative study	Type of MDTM: all Data collection method: survey *N* = 1636 surveys to MDT-members (RR 80%) Period: 2009 Country: United Kingdom	* AC		* I		
Lee Y. et al., 2017 [57]	Qualitative study	Type of MDTM: all Data collection method: survey *N* = 1000 MDT-members (RR 18%) Period:- Country: Korea	* D		* HI		
Lumenta D. et al., 2019 [58]	Observational Prospective observational study	Type of MDTM: all Data collection method: MDTM observation *N* = 27 MDTMs, 244 patients discussed Period: - Country: United Kingdom					*
Makaskill E. et al., 2006 [59]	Qualitative study	Type of MDTM: breast cancer Data collection method: survey *N* = 250 surveys to surgeons (RR 61%) Period: - Country: United Kingdom	* ACDFG			*	
Massoubre J. et al., 2018 [60]	Prospective observational study	Type of MDTM: head and neck cancer Data collection method: MDTM observation *N* = 119 patients Period: May – November 2014 Country: France	* E				
Mullan B. et al., 2014 [61]	Prospective observational Study	Type of MDTM: head and neck cancer Data collection method: MDTM observation *N* = 10 MDTMs, *N* = 105 patients Period: January – September 2011 Country: United Kingdom	* F				
Neri E. et al., 2021 [62]	Qualitative study	Type of MDTM: all Data collection method: survey *N* = 292 radiologists Period: 2018 Country: Germany	* C				
Oeser A. et al., 2018 [63]	Qualitative study	Type of MDTM: head and neck cancer Data collection method: survey *N* = 8 clinical experts Period: - Country: Germany	* C		* H		
Ottevanger N. et al., 2013 [64]	Prospective observational study + qualitative study	Type of MDTM: all Data collection method: MDTM observation ans interviews *N* = 18 MDTMs, 15 chairs interviewed Period: January 2010 – April 2011 Country: The Netherlands	* ABCDG				
Patkar V. et al., 2012 [65]	Qualitative study	Type of MDTM: breast cancer Data collection method: survey *N* = 1295 cases, 48 surveys (RR 89%) Period: - Country: United Kingdom			* H		*
Pype P. et al., 2017 [66]	Qualitative study	Type of MDTM: all Data collection method: Semi-structured interviews *N* = 16 interviews with general practitioners Period: May–June 2014 Country: Belgium	* D		* H		
Rajasekaran R. et al., 2021 [67]	Qualitative study	Type of MDTM: sarcoma Data collection method: survey *N* = 39 MDT-members (RR 92%) Period: May 2020 Country: United Kingdom	* B				
Rankin N. et al., 2017 [68]	Qualitative study	Type of MDTM: lung cancer Data collection method: survey *N* = 41 participants for documentation template development and implementation *N* = 492 surveys to general practitioners (RR 12%)	* G				
Rankin N. et al., 2018 [69]	Qualitative study	Type of MDTM: lung cancer Data collection method: structured interviews and survey *N* = 492 surveys (RR 12%), 35 interviews with general practitioners Period: May 2014- May 2015 Country: Australia	* D				
Robinson T. et al., 2017 [70]	Prospective observational study + qualitative study	Type of MDTM: all Data collection method: MDTM observation and semi-structured interviews *N* = 43 MDTMs observed, 18 interviews Period: 2013 Country: Australia	* CG		* H		*
Rosell L. et al., 2018 [71]	Qualitative study	Type of MDTM: all Data collection method: survey *N* = participants of 50 MDTMs, 362 surveys (RR 67%) Period: - Country: Sweden	* C		* HI		
Salami A. et al., 2015 [72]	Retrospective cohort study	Type of MDTM: hepatocellular carcinoma Data collection method: analyse database *N* = 116 patients in total. *N* = 48 patients discussed through virtual MDTM	* B				
Salloch S. et al., 2014 [73]	Prospective observational study + qualitative study	Type of MDTM: all Data collection method: MDTM observation *N* = 14 MDTMs, 136 cases Period: - Country: Germany			* I		
Sarkar S. et al., 2014 [74]	Qualitative study	Type of MDTM: urological cancers Data collection method: Semi-structured interview *N* = 20 MDT-members Period: - Country: United Kingdom	* BC		* HI		
Scot R. et a, 2020 [75]	Cross-sectional observational study	Type of MDTM: gynaecological cancers Data collection method: MDTM observation *N* = 41 MDT case discussions Period: 6^th^ – 29^th^ March 2019 Country: United Kingdom			* I	*	*
Shah S. et al., 2014 [76]	Prospective observational study	Type of MDTM: colorectal cancer Data collection method: *N* = 267 cases Period: - Country: United Kingdom					*
Snyder J. et al., 2017 [77]	Qualitative study	Type of MDTM: neuro-oncology Data collection method: survey *N* = 85 surveys to MDTM-chairs (RR 54%) Period: November 2015 – February 2016 Country: United States of America	* BD		* H	*	
Soukup T. et al., 2016 [78]	Cross-sectional observational study	Type of MDTM: breast cancer, lung cancer, colorectal cancer, urological cancers Data collection method: MDTM observation *N* = 1045 cases Period: 2010–2014 Country: United Kingdom	* C				
Soukup T. et al., 2016 [79]	Prospective observational study	Type of MDTM: breast cancer, lung cancer, colorectal cancer, urological cancers Data collection method: MDTM observation *N* = 1045 cases Period: 2010–2014 Country: United Kingdom			* HI		
Soukup T. et al., 2019 [80]	Longitudinal observational study	Type of MDTM: breast cancer Data collection method: MDTM observation *N* = 1355 cases reviewed Period: 2013 – 2015 Country: United Kingdom			* H		
Soukup T. et al., 2020 [81]	Cross-sectional observational study	Type of MDTM: breast cancer, gynaecological cancers, colorectal cancer Data collection method: MDTM observation *N* = 30 MDTMs, 822 cases discussions, 44 MDT-members Period: September 2015 – July 2016 Country: United Kingdom			* H		
Stone E. et al., 2018 [82]	Qualitative study	Type of MDTM: lung cancer Data collection method: survey + consensus conference (Delphi process) *N* = 350 survey’s round 1 (RR 35%), 98 surveys round 2 (RR 53%), 14 participants for consensus conference on MDTM dataset Period: - Country: Australia	* B		* H		
Stone E. et al., 2020 [83]	Qualitative study	Type of MDTM: lung cancer Data collection method: survey and interviews *N* = survey 1; 18 MDT-members, survey 2; 25 MDT-members, 6 semi-structured interviews Period: - Country: Australia					*
Taylor C. et al., 2012 [84]	Prospective observational study	Type of MDTM: colon cancer Data collection method: MDTM observation *N* = 10 MDTMs Period:- Country: United Kingdom					*
Taylor C. et al., 2012 [85]	Qualitative study	Type of MDTM: all Data collection method: survey *N* = 637 MDT-members (RR 52%) Period: - Country: United Kingdom					*
Taylor C. et al., 2021 [86]	Qualitative study	Type of MDTM: breast cancer Data collection method: interviews *N* = 36 interviews, 10 MDTMs Period: 2014 Country: United Kingdom					*
Vetto J. et al., 1996 [87]	Qualitative study	Type of MDTM: all Data collection method: survey *N* = 22 MDT-members (RR 100%) Period: 1990 – 1991 and 1992–1993 Country: United States of America	* F				
Wallace I. et al., 2019 [88]	Prospective observational study + Qualitative study	Type of MDTM: gynaecological cancers, hematological cancers, skin cancers Data collection method: MDTM observation and thematic analysis of cases *N* = 122 MDTMs, 58 cases Period: - Country: United Kingdom			* H		
Wihl J. et al., 2021 [89]	Prospective observational study	Type of MDTM: sarcoma, hepato-biliary cancer, neuro-oncology cancers Data collection method: MDTM observation *N* = 336 cases, 3 MDTMs Period: April 2019 – October 2019 Country: Sweden	* C		* I		
Wright F. et al., 2009 [90]	Qualitative study	Type of MDTM: all Data collection method: survey *N* = 385 MDT-members (RR 44%) Period: March 2007 – May 2007 Country: Canada	* D				
Yuan Y. et al., 2018 [91]	Retrospective observational study	Type of MDTM: gastro intestinal cancers Data collection method: analyse database *N* = 3674 patients discussed in MDTMs Period: January 2015 – December 2016 Country: China	* BF		* H		

^a^The theme of MDTM characteristics and logistics was divided into 7 categories: A = Schedule, B = Meeting discipline and circumstances, C = Preparation, D = Attendance, E = Patients attending MDTMs, F = Cases and streamlining, G = administrative support

^b^The Theme of decision making was divided into 2 categories: H = Decision making process and I = patient advocacy

^c^Abbreviations: *MDTM *multidisciplinary team meeting, *RR *response rate

**Table 2:** Recommendations for a high-quality oncological multidisciplinary team meeting

1. MDTMs should be routinely scheduled during working hours
2. MDTMs should have a strict meeting discipline with structured presentation of information, projected imaging results and a structured discussion without interruptions. This could be included in written team guidance
3. Ensure a clear agenda with timely availability of clinical results and protected time for the core members to prepare their cases
4. Ensure attendance of all MDTM core members
5. Establish an appropriate amount of time per case; streamlining of cases might be a way to achieve this
6. Decisions made during MDTMs should be documented, preferably by an administrative support assistant using a standardised documentation template and during the meeting
7. Pay attention to a good team culture and align tasks and responsibilities among MDT-members
8. Enable structured representation of patient characteristics and preferences by the attending physician or clinical nurse specialist during the MDTM
9. Make education an explicit goal of the MDTM for all team members and enable junior doctors to actively participate
10. The process and functioning of MDTMs require structured evaluations. Several evaluation tools can be used for this, although none of these tools have proven to optimise MDTM functioning
11. Data collected during MDTMs can be used for evaluating an MDTM’s own functioning and for additional purposes (e.g. epidemiological research) and this should be facilitated. Future developments should focus on computerized clinical support systems, to implement patient data, make guidelines-based recommendations or identify patients eligible for clinical trials

Abbreviations: *MDTM* multidisciplinary team meeting; *MDT* multidisciplinary team

### MDTM characteristics and logistics

Although there is no direct evidence that the quality and functioning of an MDTM is (at least in part) determined by its set-up, it seems clear that a well-organised MTDM is a basic requirement. Reviewing the literature, seven subcategories can be distinguished: 1) schedule, 2) meeting discipline and circumstances, 3) preparation, 4) attendance, 5) patient attendance, 6) cases and streamlining, and 7) administrative support.

#### Schedule

A factor we identified which impacts on the functioning of MDTMs is a clear meeting schedule with protected time within working hours. In 2013 Ottevanger et al. observed 18 MDTMs in seven Dutch hospitals and interviewed the chairpersons. Adherence to the weekly MDTM schedule was found to be a precondition for an effective MDTM, which was the case in 100% of the tumour-specific MDTMs (*n* = 14) and in only 40% of the general oncological MDTMs (*n* = 5) [64]. A survey of 136 surgeons participating in breast cancer MDTMs reported that only 28% of MDTMs were held during regular working hours [59]. A majority of the participants suggested dedicated time for MDTMs during working hours as an improvement [59]. When time for MDTMs is set aside in the participants’ working schedule, their personal contributions to MDTMs improve [31, 51, 56].

#### Meeting discipline and circumstances

Meeting discipline and interruptions affect the efficacy of MDTMs. Interruptions during MDTMs seem to be common. On average 6 to 11 people were walking in and out of the MDTM and 4 to 6 phone calls disturbed the meeting, according to Ottevanger et al [64].

Structured information presentation, projected clinical imaging results, structured case discussions and written team guidance have been shown to improve ability to reach a multidisciplinary team decision and improves the quality of the information presented [31, 47, 53, 54, 63, 74, 77, 82, 91]. Conference call or video conferencing is becoming the standard of care, as it facilitates the attendance of highly specialised clinicians, minimises travel time and reduces the duration of the diagnostic trajectory [25, 26, 72, 91]. Thirty-six sarcoma MDT members were obliged to participate in completely virtual MDTMs due to the COVID-19 pandemic: 73% were satisfied with the depth of the discussion and 83% felt that decision making had not changed following the switch from face-to-face MDTMs to virtual MDTMS [67]. However, the failure of technological equipment impacts MDTMs negatively [45, 51] and the number of patients discussed per MDTM has been reported as having decreased compared to face-to-face MDTMs [26].

#### Preparation

Good preparation of an MDTM implies a clear list of patients to be discussed, timely availability of all clinical information including imaging and pathology results, and sufficient time for all core members to prepare their cases [64]. Although an MDTM agenda was nearly always present (93%), a clear presentation of a question to be discussed per patient was available in only half of the meetings (47%) in a MDTM observational study [64]. Time to prepare an MDTM has been suggested as an improvement in several studies [51, 56, 59, 74, 77]. A survey of 292 radiologists found that only 114 respondents (44%) review over 70% of cases prior to the MDTM, mainly due to lack of time [62]. In 5% of the cases discussed at general or tumour-specific MDTMs, pathology or radiology results were absent [64]. Inadequate or absent information about radiology and pathology results proved to be a barrier to making clinical decisions within the MDTM, as was a lack of up-to-date information about the patients’ comorbidities and condition [31, 45, 54, 55, 71, 78, 89]. According to two interview studies, imaging technology and real-time data support and enhance clinical discussion during MDTMs [47, 70].

#### Attendance

Attendance of core MDT members and a well-functioning chairperson are essential for clinical decision making within the MDTM [31, 55]. Several studies scored the attendance rates of the core MDT members (defined as: surgical oncologist, medical oncologist, radiologist, pathologist, radiation oncologist and an organ-specific specialist) [57, 59, 64, 90]. The results ranged from attendance rates of 49% to over 90% [57, 59, 77]. Several other studies identify non-attendance of core MDT members as a negative factor for efficient decision making during MDTMs [59, 51, 45]. Attendance of the general practitioner (GP) was examined in a semi-structured interview study of 16 Belgian GPs [66]. GPs perceived attendance at an MDTM as part of their work and benefit of the MDTM discussions and the interprofessional collaborative relationships [66]. In an Australian study, a standardised template reporting MDTM findings back to GPs was found to be a feasible alternative [68].

#### Patients attending MDTMs

A questionnaire completed by 429 breast cancer MDT members and 135 patient advocates was performed by Butow et al [22]. Only 12 health professionals (4%) reported that their work setting allowed patients to attend their MDTM. Patient advocates reported that they were not invited to attend MDTMs and only 47 (35%) was informed when their case was discussed in a MDTM. The common reasons for supporting patient involvement included patients being more informed and empowered, and to facilitate shared decision-making and improve communication between the patient and the medical team [22]. In general, healthcare providers fear attendance would increase anxiety, undermine the doctor-patient relationship (through complex discussions in jargon with different viewpoints put forward by the attending professionals) and have a negative impact on the dynamics of the meeting [22]. The effect of the presence of the patient during the MDTM, including physical examination, did not change the therapeutic decision in a prospective study among 119 head and neck cancer patients [60]. From a patient’s point of view, increased anxiety or depression due to MDTM participation was not noted in most studies, while being better informed and able to present their own preferences were named as advantages [20, 23, 24, 27, 60].

#### Cases and streamlining

In a prospective observational study of 298 urological cancer patients being discussed in seven MDTMs, cases discussed towards the end of meetings were associated with lower rates of decision-making, information quality and teamworking [55]. In addition, more available time per case was associated with improved teamworking [55]. The amount of time per case differs [35, 61, 91]. For example, an observational study of 10 head and neck cancer MDTMs found that discussion time per patient ranged from 15 s to 8 min, with a mean of 2 min [61].

A Dutch interview study of two collaborating head and neck cancer MDTMs found that only in a minority of discussed cases (8/336) was the additional value of the collaboration acknowledged and the national obligation to discuss all patients was felt to be outdated [43]. The selection of patients to be discussed in an MDTM – nowadays called ‘streamlining’ – was a topic as far back as 1996, when Vetto et al. compared the discussion of all patients in a ‘working conference’ with only discussing ‘fascinating cases’. Of 22 participants surveyed, 77% preferred to discuss all patients [87]. In 2005, a questionnaire involving 136 breast cancer MDT members showed that the selection of cases for discussion was made by the surgeon in 49%, by the medical oncologist in 34%, and by the pathologist in 25% of cases. In half of meetings, all patients were presented [59]. A questionnaire completed by 16 neuro-oncology MDT members reported a mixed response regarding which patients should be discussed: 44% (*n* = 7) thought all patients should be discussed and 56% (*n* = 9) thought only those patients with complex management issues should be discussed [34]. A recent national survey of 1220 MDT members in the United Kingdom included a question on how to enhance the effectiveness of MDTMs. They defined streamlining of discussions as follows: ‘specialist time is focused on those cancer cases that don’t follow well-established clinical pathways, with other patients being discussed more briefly’. The majority of participants (69%) agreed that streamlining could allow more straightforward cases to be dealt with more quickly and over half (60%) thought that some form of streamlining would be beneficial for their MDTM, while 25% did not think that streamlining would be beneficial [42].

#### Administrative support

Support by a coordinator or administrator was available in a minority of oncological MDTMs while a lack of administrative support was found to be a barrier to effective functioning [31, 47, 64, 70]. In a postal survey of breast cancer MDTMs in the UK, 6% of surgeons who responded noted no recording of decisions made in the MDTM [59]. Another survey of 265 MDTM coordinators reported that most of them were trained in data management and IT skills to facilitate MDTMs [44].

Interestingly, where several treatment options for the patient were discussed during an MDTM, only a single option was documented at the end of the discussion [37]. Several studies agreed on the importance of a standardised documentation template and the standard supply of a copy to the general practitioner [33, 34, 68].

### Team culture

Attending MDTMs has been reported to result in more interactive and closer working relationships between healthcare professionals of different disciplines [27, 31]. The MDTM process was seen as a peer-review process providing checks and balances and discouraging the conduct of inappropriate or unnecessary investigations. Furthermore, clinicians felt the MDTM provided some medico-legal protection [27, 57]. A focus-group study by Fahim et al. (2020) identified several barriers to team dynamics that negatively affect the decision-making process, including lack of soft skills (effective communication, collaboration), negative group dynamics (bullying), lack of psychological safety (the ability to ask questions or make mistakes), and the presence of participants who dominate the conversation [31]. According to a focus group study with allied health professionals, they often felt inhibited when offering their contribution, despite the fact that they are supposed to supply information about the patients’ condition and preferences. In their experience, there was insufficient time and respect for their information [27]. Some MDTMs were seen as intimidating and part of an ‘boys’ club’. It was easier to contribute when invited by another member of the MDT [27]. Two studies suggested a role for the chairperson in inviting all team members to contribute and arrive at case consensus [35, 51]. The setting of the MDTM has a clear influence on team culture. Face-to-face MDTMs were found to be more informal, spontaneous and conducive to open discussion. In contrast, the videoconferences were formal and regimented, appearing to reflect pre-existing hierarchical positions [26]. An interview study with 22 participants found that video-conferencing has a negative influence on decision making due to poor communication, causing conflicts and friction within the MDT [45]. In contrast, Kunkler et al. reported no differences in Group Behaviour Inventory (GBI) scales between face-to-face and video conferences [50]. Aligning tasks and responsibilities between MDT members is also important. A survey of 58 breast cancer MDT members revealed which expectations each member had regarding their own responsibilities and tasks, and those of other team members, showing discrepancies leading to gaps of information and impaired decision making [48]. The nurse specialist was the least visible of the participants. It was recommended that teams be trained to work together, especially with regard to communication skills, to ensure that patients receive comprehensive and consistent information [48].

### Decision making

Two subcategories were identified within the theme of decision making: decision-making process and patient advocacy.

#### Decision-making process

A qualitative observational study of two centres where 106 cases were discussed analysed the actual decision-making process within the MDTM [12]. They identified different sources of authority that are used to justify actions within discussions: encountered, technological, research evidence, lived experience, interpreter and referral authority. Where there was conflicting authority, encountered authority (authority based on knowing the patient) and clinical experienced authority were decisive in the decision-making process [12]. An interview study involving 179 MDT members found that where opinions were split, in 69% of cases the physician in charge of the patient made the final decision [57]. A smaller study also identified a role for the chairperson in this case [74]. In an interview study of 21 MDT members, 27 barriers were identified that hindered the decision-making process. Most commonly described barriers included gaps in leadership, lack of preparation, unstructured case presentation, individual treatment preferences of treating physicians and prolonged case discussions [31]. They also described 13 facilitators of clinical decision making, including adequate knowledge of guidelines and recent evidence, standardisation of decision making and facilitation of collegiality and teamworking [31]. An observational study noted five ways in which the clinical nurse specialist (CNS) positively contributed to the decision-making process: sharing information, asking questions, providing practical suggestions, framing and using humour [88].

Other identified factors influencing the decision-making process were ‘decision-making fatigue’ during prolonged MDTMs, time-workload pressure, logistic complexity, gender imbalance in the team and negative interactions between team members [80, 81]. ICT tools to support clinical decision making during the MDTM have been developed. In 1295 breast cancer patients a tool of this kind called MATE was found to select up to 61% more cases for clinical trial recruitment and resulted in more concordance with clinical practical guidelines [65].

#### Patient advocacy

Attending physicians, clinical nurses, patients themselves or patient advocates can represent the patient’s perspective. Clinicians see their role as being an advocate for patients discussed at MDTMs and the absence of the clinician in charge is a negative factor for the functioning of an MDTM [27, 71, 74]. An observational study of 15 MDTMs concluded that individual patient characteristics or patient treatment preferences were rarely considered or discussed and that physicians based their decision making on medical information. In the few cases where patient preferences were raised as a topic, this information did not seem to be taken into account in the decision-making processes [37]. Similar results have been reported, including MDT discussion focusing on the only recommended treatment option, or presenting the treatment option selected within the MDTM as the only option to a patient afterwards, ignoring other equally valid options discussed [20, 39]. When comparing observation with a self-assessment tool, case histories and radiological information were best presented and patients’ views and comorbidities/psychosocial issues were least well presented in a study involving 164 cancer patients and 67 team members in 5 MDTMs [52]. Other studies reported similar findings, all concluding a lack of patient centeredness in terms of available information on comorbidities and preferences [19, 21, 38, 45, 73, 79, 89]. A large survey including 1636 MDT members recommended a crucial role for the CNS in representing patient preferences [56]. A more prominent role for the CNS has also been suggested by others [75, 88]. In an observational study of 171 elderly patients with colorectal cancer a suboptimal decision-making process was observed due to limited use of patient-centered information, such as age-related patient characteristics and preferences [21]. It was found that remarks about general condition in terms of vitality or frailty were significantly more often mentioned during the MDT discussion when a participant with geriatric expertise was present (11% vitality and 19% frailty) compared to an MDTM without geriatric expertise (3% and 8% respectively) [21].

### Education

To ensure that MDTMs continue to function well in the near future, it is important to train junior doctors to participate in MDTMs, on the basis of the master and apprentice principle. Up to three quarters of surgeons participating in breast cancer MDTMs saw an educational opportunity for trainees at their MDTMs, according to a survey of Macaskill et al [59]. Eighty-seven percent of 45 neuro-oncology participants agreed that education of fellows, residents and students in MDTMs is a value point [77]. In a survey study including 72 MDTM chairpersons the permission rate to attend the MDTM was 76% for residents and 39% for medical students [28]. An observational study of 52 gynaecological MDTMs noted that fellows and residents were expected to prepare cases in advance to gain a clearer understanding of the subtleties of care from the academic discourse. Educational case discussion focused primarily on treatment options and planning [36]. Junior doctors, trainees and medical students are frequently seated in the outer circle, away from the inner circle where the discussion mostly takes place [47]. Junior physicians were not observed to play a prominent role in the decision-making process and were sometimes asked to perform tasks during the meeting, preventing adequate participation [37, 47, 75]. In four focus groups with 23 participants the educational benefit of attending MDTMs was noted. Nurses and allied health professionals appreciated the opportunity to view pathology and radiology results and achieved a greater understanding of medical care and the decision-making process. Junior medical staff did not participate in the focus groups but were thought to benefit from witnessing decision making by senior staff in determining a treatment plan [27]. Another focus group study with 18 participants recognised the educational benefit of MDTMs in enhancing knowledge or understanding [31]. A Spanish nationwide survey found that in 33 out of 71 MDTMs, educational activities were organised once a year for all MDT members [28].

### Evaluation and data collection

Up to nine MDTM evaluation tools aiming to improve the functioning of MDTMs have been identified and are summarised in Table 3**.** The MDT-MOT tool was originally developed by Lamb et al., [52] and then adapted by Jalil et al., [46] Shah et al., [76] and Harris et al [41]. Table 3 shows this together. Most of these tools only score items that have been reported to be essential for a well-functioning MDTM [5, 6]. In seven of the nine tools predefined items were scored by an observer [40, 41, 46, 49, 52, 76, 84], while two tools are a self-assessment measure [29, 85]. The categories scored are most commonly the more practical items such as attendance, availability of all patient data, and organisation and administration of the MDTM. Six tools evaluate the performance of the chairperson [29, 40, 41, 46, 49, 76] and three tools evaluate the team culture [40, 41, 85]. Personal development and training are explicitly scored in the tool of Harris et al. [40]. and mentioned in the self-assessment tool of Taylor et al [85]. The perspectives and preferences of the patient are explored in the tools of Evans et al. [29] Jalil et al. [46] Shah et al. [76] and Taylor et al [84]. The education and training function of MDTMs is explored in four tools [29, 40, 84, 85] The tool of Evans et al. is the only one that measures communication with the general practitioner [29]. In this tool a ‘maturity score’ of the MDT was determined on the basis of five domains based on self-assessment [29]. In a follow-up study, results from three years of self-assessment (2017–2019) of 12 MDTs were compared; in nine out of 17 questions a significant improvement was observed. Highly significant improvements were seen for documenting consensus, developing terms of reference, referring to clinical practical guidelines, and establishing referral criteria. There was no significant change for questions related to patient considerations, professional development and quality improvement activities [30]. In a before and after study design, the implementation of up to five interventions to optimise decision making (use of discussion tools, workshops, MDT or chairperson training, audit and feedback) was evaluated using two of the mentioned tools (MTB-MODe and MDT-OARS). Four MDTs were evaluated before and after the implementation of a mean of three interventions. The quality of the per-case decision making did not improve significantly (*P* = 0.78) [32]. The tools MDT-OARS and TEAM were further developed into the ‘MDT feedback for improving teamwork (MDT-FIT)’ programme and implemented in 10 breast cancer MDTMs in the UK. This programme consists of 3 stages (set-up, assessment, feedback including actions for improvement) and lasts for 8–12 weeks. Within 36 interviews the acceptability, appropriateness and feasibility of MDT-FIT was found to be moderate to high [86]. Results of MDT-FIT are lacking. Several other studies evaluated the quality of teamworking by using the MTB-MODe tool and found this manner of direct observation feasible and reliable [35, 58]. In summary, there are several feasible evaluation tools available that are useful in guiding the evaluation process, however none of them have yet proven to optimise MTDM functioning. In MDTMs, structured and multidisciplinary data is collected, which can be used to further improve care and to evaluate an MDTM’s own functioning. In 15 semi-structured interviews, participants were unanimous that data collection during MDTMs was important and should be enabled by health information systems. They also expressed concerns about the quality of data that was currently collected through MDTMs [47]. A study that evaluated a self-assessment tool concluded that nine out of 117 respondents confirmed that internal audits were performed to assess whether treatment decision making made in the MDTM was in line with current best practices [29]. Robinson et al. performed an ethnographic study on engaging MDTMs in translational research and quality improvement and found that the capture of real-time data was a priority in helping involve teams more actively in quality-improvement activities [70]. A mixed method survey, interview and observational study on the impact of data collection in three lung cancer MDTMs found that data regarding number of cases, stage, final diagnosis and time to diagnosis and treatment was collected. This data was found to be easy to interpret and relevant for both clinical practice and the MDTM [83].

**Table 3:** Multidisciplinary team meeting evaluation tools

Name of Evaluation tool	Abbreviation	Execution	Scoring domains	Author
Multidisciplinary team maturity matrix	-	An annual member survey and a self-assessment tool to monitor team performance 5 domains are scored in 5 performance levels (basic to advanced, maximum matrix of 100 points)	Governance and leadership (including leadership, obligations of team members, decision-making, risk management) Meeting organization and logistics (including logistics and representation, pre-meeting, at meeting, post-meeting) Linkages and communication with general practitioners (GP’s) and patients (including access for GP’s, communication with GP’s (patients), information and education (general), patients) Data collection, analysis and research (including data collection, monitoring and evaluation, data quality and system integration, research) Infrastructure and human resources (including facilities and equipment, MDT co-ordination, care co-ordination, data management)	Evans L. et al., 2019 [29]
Team Evaluation and Assessment Measure	-	10 subdomains scored by observers each on a 10-point scale (1 very poor, 10 very good), including comments	Attendance, Leadership and chairing, Teamworking and culture, Personal development and training, Physical environment of meeting venue, Technology and equipment availability, Organization and administration, Post-meeting co-ordination of services, Patient-centered care, Clinical decision making processes	Harris J. et al., 2014 [40]
Multidisciplinary team meeting observational tool	MDT-MODe; after adjustments MDT-MOT	3 domains of teamworking scored by observers each on a 5-point scale (1 very poor, 5 very good)	Attendance Leadership/chairing of the MDT meeting Teamwork and culture	This tool was originally developed by Lamb B. et al., 2011 [52], adapted by Jalil R. et al., 2014 [46], Shah et al., 2014 [76] and lastly by Harris J. et al. 2016 [41]
Peer review framework	-	8 domains to assess MDT performance by peer-reviewers, scoring ‘under developed / developing / well developed’	Structure and governance Membership and leadership Meeting organization and support Standards of care Patient involvement Quality assurance Professional development Financial governance	Johnson C. et al., 2017 [49]
Team evaluation and assessment measure	TEAM	Multidisciplinary team self-assessment tool; survey with 47 items on 5 domains (17 subdomains) to be rated on 5-pont scale	Team (membership, attendance, leadership & chairing, teamworking & culture, Personal development & training) Infrastructure of meetings (physical environment of meeting venue, technology & equipment) Organization and administration for meetings (scheduling, preparation, prior to meetings, organization/administration during meeting, post-MDT meeting coordination of service) Patient-centered clinical decision making (who to discuss?, patient-centered care, clinical decision making process) Team governance (organization support, data collection, analysis & audit of outcomes, clinical governance)	Taylor C. et al., 2012 [85]
Multidisciplinary team observational assessment rating scale	MDT-OARS	4 domains with 15 aspects of MDT working scored by observers on a 4-point scale (very poor – very good)	Team (attendance, leadership; chairing, teamworking & culture, personal development & training) Infrastructure for meetings (meeting venue, technology & equipment) Meeting organization and logistics (preparation prior to meetings, organization/ administration during meetings) Clinical decision making (patient centered care, treatment plans) Characteristic of effective MDT-working (teamwork & culture)	Taylor C. et al., 2012 [84]

## Discussion

This extensive systematic literature review identified five themes (MDTM characteristics and logistics, team culture, decision making, education and evaluation and data collection) that are important for an efficient, well-functioning and high quality MDTM and results in feasible recommendations (Table 2). A clear and structured meeting schedule is a prerequisite, attendance of all core MDTM members mandatory, as well as sufficient preparation time, especially for radiologists and pathologists, if review of the investigations is desired. Clear formulation of the question to be answered may be helpful in the decision-making process. Technical and administrative support, the latter not only for preparation but also for reporting, is a boundary condition. A regular evaluation based on plan/do/check/act is necessary to see if all goals have been met. Small adjustments to improve these elements can already result in a significant improvement in the quality of MDTMs. Team skills, such as effective communication and collaboration are important, therefore team training is suggested [31]. We did not identify any literature regarding personal competences or skills and their impact in the functioning or quality of MDTMs.

Although MDTMs are organised in the interests of patients, the latter are seldom present at the meeting where their case is discussed. In general, healthcare providers take a cautious attitude to patient participation. They fear it may cause anxiety and undermine the doctor-patient relationship, and some doctors do not want to confront patients with conflicting opinions about the best treatment [20, 22, 27, 60]. For some patients it might be disappointing to witness their cancer diagnosis, which turned their lives upside-down, being discussed in only a few minutes. On the other hand, the majority of patients report feeling better informed without suffering increased anxiety [22–24] Massoubre et al. compared therapeutic decision-making in the MDTM following discussion of a patient file, or after patient participation in the MDTM, and found a concordance rate of 97% [60]. They concluded that the patient’s presence was not essential, provided the medical file was complete and current. An advantage of the absence of the patient was that it decreased the duration of the meeting [60]. On the basis of the literature, it is impossible to provide a definitive recommendation. Nevertheless, it remains an important issue. Well defined studies are needed to answer these questions.

MDTMs are currently under pressure due to the increasing number of patients that need to be discussed in relatively little time [4]. Streamlining of cases can reduce the pressure on MDTMs. A distinction can be made between standard and complex cases: only complex cases would be discussed in the MDTM while standard cases would be handled on the basis of predetermined guidelines/algorithms [92, 93]. Support for streamlining varied considerably in the different studies we identified [34, 42, 43, 87]. Considering the MDTM as a means of medico-legal protection might give cause for hesitation [27, 57]. Another disadvantage of case selection is the lack of opportunity to use the MDTM as source for collecting patient data for research or quality improvement purposes [29, 47, 70, 83]. However, we believe that given the context of increasing cancer incidence and prevalence, and the development of more complex and new multidisciplinary treatment options, streamlining is inevitable in the near future. That said, further research on patient case selection is needed and alternative methods of data collection must be explored.

In COVID-19 times, MDTMs came under pressure due to a high working load of most MDT members, the emotional impact of COVID-19 care on health care professionals, decreased availability of diagnostic and treatment facilities and simply restrictions in the number of persons allowed in rooms or accelerated turning to digital MDTMs. Despite this, team skills seemed not to be much affected by the COVID-19 pandemic, since the depth of discussions did not change following the switch from face-to-face MDTMs to virtual MDTMs [67]. Furthermore, Grosclaude et al. (2020) measured the MDTM activity of 191 different French MDTMs in the period of January 2019 – April 2020 and found only a moderate decrease of 8% less meetings during this period, which reflects the commitment of these teams to MDTMs [94]. The decision-making process in MDTMs is influenced by a large number of factors. Knowing the patient, clinical experience and leadership contribute most to actual decision-making [12, 27, 31, 57, 71, 74]. Patients presented in the MDTM by their attending physician were up to 20% more likely to receive a correct diagnosis [95]. Remarkedly, multiple studies have found a lack of patient centeredness in the MDTM (i.e. insufficient knowledge of patient preferences and comorbidities during the discussion) [19–21, 27, 37–39, 45, 52, 59, 71, 73, 79, 89]. A crucial role was seen for the CNS in representing patient preferences [56, 75, 88]. Further steps are needed to improve the patient centeredness of MDTMs, for example explicit mention of patient preferences in the registration form or introduction of a dedicated representative (i.e. attending physician or CNS) who participates in the MDTM. Although we realize that being able to have a patient representative present for every patient case has many practical challenges.

The NCAT 2010 guidelines state ‘There is a teaching and training role for MDTs both within the team itself (e.g. bringing patient cases back) and beyond (e.g. for clinicians in training)’ [6]. Although the educational function of an MDTM has been acknowledged [27, 31, 59, 77], implementation in practice seems to be difficult. Junior doctors do not have an active role within the MDTM. For them the process was believed to be passive: mainly observation of decision-making by the senior staff [27, 31, 36, 37, 47, 75]. The focus of the learning process was on medical competences. No literature was found on the educational function of the MDTM for junior doctors, as well as core members, regarding other competences such as collaboration and communication. In any event it is important to state whether education of junior staff is also the aim of the MDTMs. In that case education tools have to be well-defined and incorporated in the evaluation cycle.

Many tools have been developed to evaluate the functioning of MDTMs. Most of these tools are used by an observer who scores the predefined (predominantly practical) items during an MDTM. The feasibility of the MTB-MODe, MDT-OARS and TEAM tools as well as the MDT-FIT programme has been demonstrated but their impact on improving the functioning of MDTMs remains unclear.

Future developments to further improve the quality of MDTMs include the use of computerised clinical support systems (CDSSs), which implement patient data, make guidelines-based treatment recommendations or identify patients eligible for clinical trials [93, 96, 97].

### Strengths and limitations

Our systematic review has several strengths. Two independent authors meticulously searched over 4,700 articles, and a third author was included in the event of conflicting judgements. This article therefore presents a complete overview of known factors influencing the quality and functioning of MDTMs and makes recommendations on optimising MDTMs for healthcare providers. Due to the heterogeneity of studies that we reviewed, a fully methodological review according to the PRISMA guidelines [98] was not feasible. However, all full text articles have been reviewed for both relevance and quality by three independent researchers (JW, OvdH, ID) as good as possible. Furthermore, on account of the lack of formal evidence-based criteria guaranteeing high-quality MDTM functioning, assumptions have sometimes been made. The study period spans three decades. Some results from older literature (e.g. ICT problems) may no longer be a problem at this point in most countries. However, it does reflect the conditions that a high-quality MDTMs must meet. In order to obtain a complete overview of all literature on the optimal functioning of MDTMs, an inclusion date from 1990 has been chosen, as that was the time when MDTMs were first introduced in cancer care. However, we realize that some items have been resolved, and new ones, for example the enormous number of patients to be discussed, became more important.

## Conclusion

In this systematic review we show that, in addition to a more structured meeting and the presence of all MDTM core members, there should be sufficient discussion time for all cases with more emphasis on patient centeredness. Streamlining of cases and training the MDT could be the way to achieve this.

## Supplementary information

**Additional file 1:** **Supplement A** – Full description on the executed search

## Data Availability

All data generated or analysed during this study are included in this published article and its supplementary information file.
