# Supplementary material for: Factors influencing the quality and functioning of oncological multidisciplinary team meetings: results of a systematic review
Source: BMC Health Serv Res. 2022 Jun 27;22:829. doi: 10.1186/s12913-022-08112-0 (PMC9238082; doi:10.1186/s12913-022-08112-0)
Supplement: Supplementary file 1 — Additional file 1: Supplement A – Full description on the executed search [file 12913_2022_8112_MOESM1_ESM.docx]

**Supplement A – Full description on the executed search**

Included databases: Medline, Embase and PsychInfo

Search terms for oncological multidisciplinary team meetings, including in title or abstract:

cancer board(s) OR Cancer conference(s) OR cancer meeting(s) OR cancer multidisciplinary team(s) OR case conference(s) OR case discussion(s) OR case presentation(s) OR case review(s) OR clinical conference(s) OR clinical meeting(s) OR clinical team(s) OR interdisciplinary conference(s) OR interdisciplinary meeting(s) OR interdisciplinary team(s) OR multidisciplinary cancer care OR multidisciplinary cancer conference(s) OR multidisciplinary cancer team(s) OR multidisciplinary clinic(s) OR multidisciplinary communication OR multidisciplinary conference(s) OR multidisciplinary consultation(s) OR multidisciplinary discussion(s) OR multidisciplinary meeting(s) OR multidisciplinary review(s) OR multidisciplinary team(s) OR multidisciplinary team management OR multidisciplinary team meeting(s) OR Multidisciplinary teamwork OR multidisciplinary tumor board(s) OR multidisciplinary tumour board(s) OR multidisciplinary tumour board meeting(s) OR oncology board(s) OR oncology conference(s) OR oncology meeting(s) OR tumor board(s) OR tumor conference(s) OR tumor meeting(s) OR tumour board(s) OR tumour conference(s) OR tumour meeting(s).

OR

Mesh terms (Medline)

Patient care team OR interdisciplinary studies OR interdisciplinary communication

AND

Mesh term (Medline) / Emtree term (Embase and PsychInfo)

Neoplasm

AND

Limits

1-1-1990 – 1-1-2019

English language

No conferences, erratum, books, chapters or encyclopedias

Total hits found

|  | Number of hits  ( n =) |
| --- | --- |
| Medline | 2048 |
| Embase | 2294 |
| PsychInfo | 36 |
| Total | 4378 |
| Number of duplicates | 581 |
| **Number of records included** | **3797** |
